# Phytochemical Diversity and Antioxidant Potential of Natural Populations of *Nelumbo nucifera* Gaertn. throughout the Floristic Regions in Thailand

**DOI:** 10.3390/molecules27030681

**Published:** 2022-01-20

**Authors:** Duangjai Tungmunnithum, Samantha Drouet, Christophe Hano

**Affiliations:** 1 Department of Pharmaceutical Botany, Faculty of Pharmacy, Mahidol University, Bangkok 10400, Thailand; 2 Laboratoire de Biologie des Ligneux et des Grandes Cultures, INRAE USC1328, Campus Eure et Loir, Orleans University, 28000 Chartres, France; samantha.drouet@univ-orleans.fr; 3 Le Studium Institute for Advanced Studies, 1 Rue Dupanloup, 45000 Orleans, France

**Keywords:** phytochemical profiles, antioxidant potential, *Nelumbo nucifera*, sacred lotus, Nelumbonaceae, population level, floristic regions

## Abstract

Asian lotus has long been consumed as a food and herbal drug that provides several health benefits. The number of studies on its biological activity is significant, but research at the population level to investigate the variation in phytochemicals and biological activity of each population which is useful for a more efficient phytopharmaceutical application strategy remains needed. This present study provided the frontier results to fill-in this necessary gap to investigating the phytopharmaceutical potential of perianth and stamen, which represent an important part for Asian traditional medicines, from 18 natural populations throughout Thailand by (1) determining their phytochemical profiles, such as total contents of phenolic, flavonoid, and anthocyanin, and (2) determining the antioxidant activity of these natural populations using various antioxidant assays to examine different mechanisms. The result showed that Central is the most abundant floristic region. The stamen was higher in total phenolic and flavonoid contents, whereas perianth was higher in monomeric anthocyanin content. This study provided the first description of the significant correlation between phytochemical contents in perianth compared with stamen extracts, and indicated that flavonoids are the main phytochemical class. This analysis indicated that the stamen is a richer source of flavonoids than perianth, and provided the first report to quantify different flavonoids accumulated in stamen and perianth extracts under their native glycosidic forms at the population level. Various antioxidant assays revealed that major flavonoids from *N. nucifera* prefer the hydrogen atom transfer mechanism when quenching free radicals. The significant correlations between various phytochemical classes and the different antioxidant tests were noted by Pearson correlation coefficients and emphasized that the antioxidant capability of an extract is generally the result of complex phytochemical combinations as opposed to a single molecule. These current findings offer the alternative starting materials to assess the phytochemical diversity and antioxidant potential of *N. nucifera* for phytopharmaceutical sectors.

## 1. Introduction

*Nelumbo nucifera* Gaertn. is an aquatic perennial plant species belonging to the Nelumbonaceae family [1,2,3,4,5]. This plant is also commonly known by its common names, e.g., lotus, sacred lotus, Asian lotus, or Indian lotus [1,2,3,5,6]. *N. nucifera* is mainly distributed in tropical and subtropical regions of Asia, such as Thailand, China, Napal, India, and other Asian nations. This lotus species was also introduced to some countries in the European Union, such as Romania and Hungary [7].

*N. nucifera* is well-recognized for its beautiful flowers, especially the color, size. and morphology of its perianth (petal). This sacred lotus has also been considered as a spiritual symbol for Buddhists and Hindus since the ancient periods. Furthermore, *N. nucifera* also offers many health benefits to mankind. For example, its perianth and rhizomes are used as vegetable ingredients for various type of cuisines. Additionally, its seeds can be consumed as fresh fruit. Besides, many parts of its flower are also employed in important recipes for traditional medicines or herbal drugs, especially in Thai traditional medicines and Chinese traditional medicines [5,6,8,9,10,11]. The perianth and stamen are also used to prepare herbal teas for relaxation and healthy benefits [5,6,9].

During the last two decades, the number of research studies to evaluate the potential biological activities of *N. nucifera* have considerably increased [8,9,10,11,12,13,14,15,16,17]. Several parts of this lotus species have been investigated, i.e., the antioxidant effect from stamen methanolic extract [12], the seed hydroalcoholic extract [18], epicarp extract [15], embryo extract [16,19], as well as leaf extract [8,11]. In addition, the anti-obesity activity [20], anti-inflammatory activity [21,22], enhancement of sleeping quantity and quality [23], potential to improve muscle wasting [24], as well as UV-protection and anticancer potential [25] of *N. nucifera* leaf extract were also reported by previous research works. The therapeutic effect of *N. nucifera* methanolic extract on pathological cardiomyocyte hypertrophy was also evaluated [26]. The anti-Alzheimer’s disease potential of the aqueous extract of the mixture from leaf, flower (petal and stamen), petiole, peduncle, and seed embryo of *N. nucifera* was also studied [27]. According to the previously published works, it is obvious that there is no research study that examined the biological activity or phytochemical profiles of *N. nucifera* at the population level. Nevertheless, the great variation among several populations in each plant species, which may possibly lead to the variation in phytochemical profiles and effect on biological activities, should be taken into account [28,29]. Moreover, Thailand is one of the important hot spots of species biodiversity, including *N. nucifera* plants. 

The present study aimed to study the variation in phenolic compounds (phenolics, flavonoids, and anthocyanins) and antioxidant properties of stamen and perianth extracts of 18 *N. nucifera* populations from different geographical regions in Thailand. The goal of this study is to complete this knowledge by determining the total phenolic, flavonoid (including HPLC determination of the main flavonoids), and anthocyanin contents as well as antioxidant activity using several in vitro assays based on different mechanisms, with 18 natural populations originating from all Thai floristic regions.

## 2. Results and Discussion

### 2.1. Plant Populations and Botanical Description

According to the field study and the intense search for living plant specimens of *N. nucifera* in the fields, 18 populations of this sacred lotus species were collected from different localities (provinces) covering all seven floristic regions in Thailand, as shown in the Table 1. 

**Figure 1:**
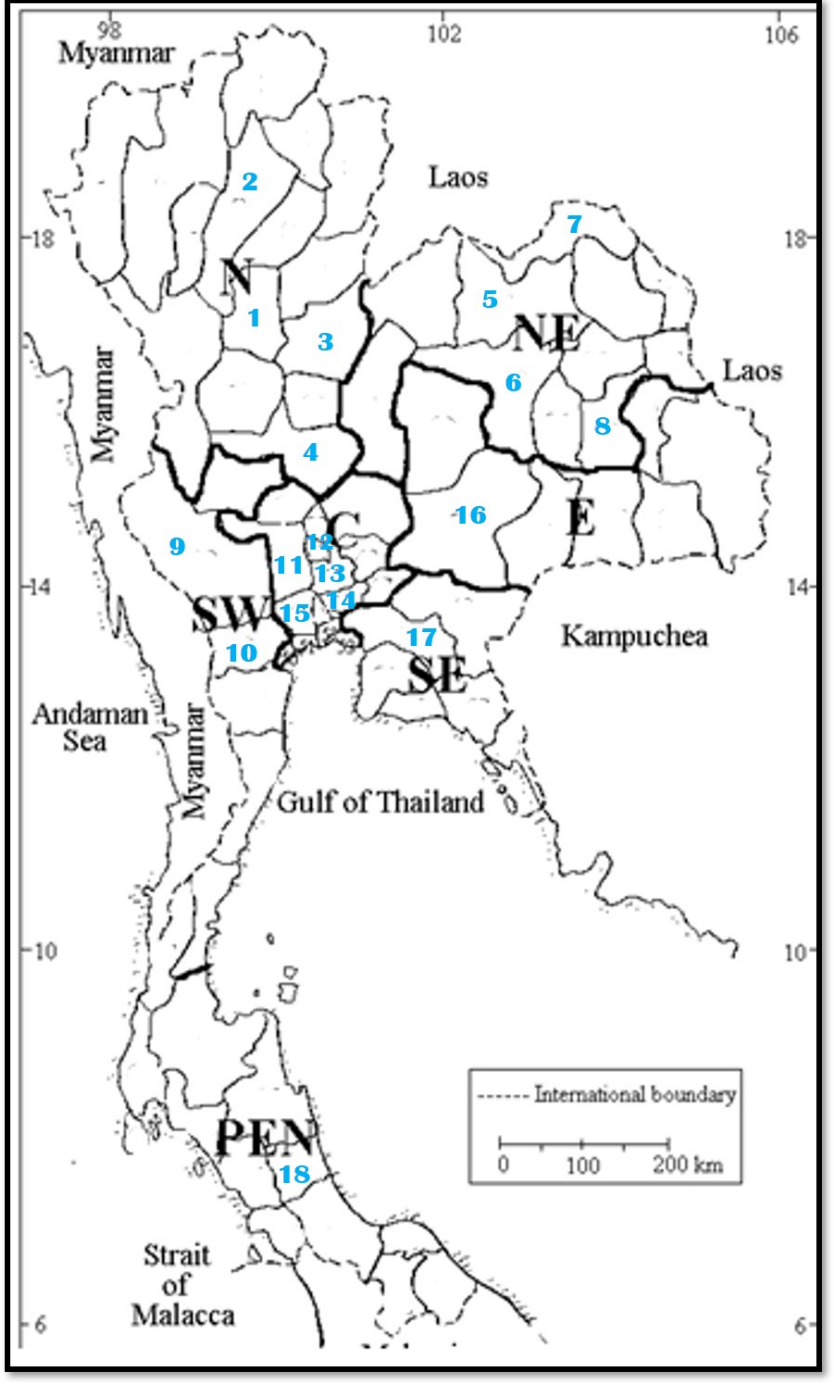
The distribution map of *N. nucifera* 18 populations collected from the natural habitat cover the floristic region in Thailand. The number 1–18 in the map indicates the population number.

**Figure 2:**
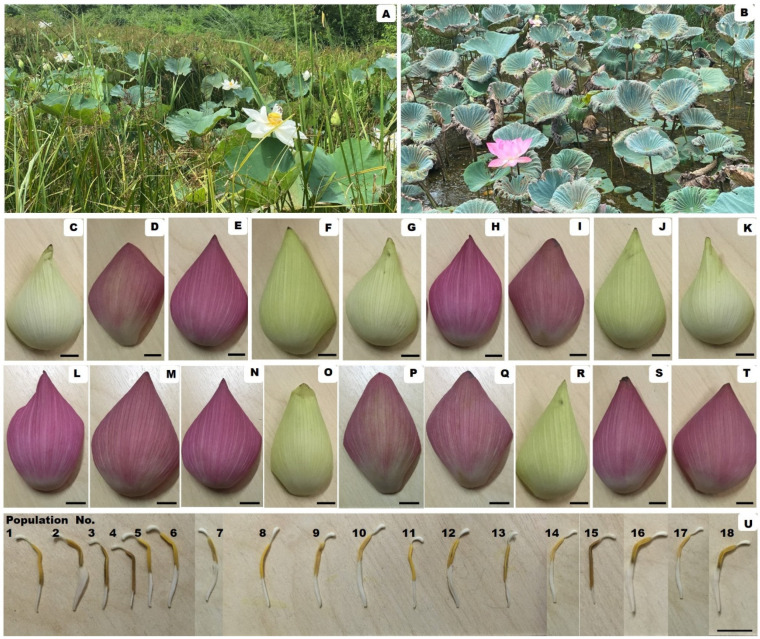
*N. nucifera*: (**A**,**B**). Natural habitats; (**C**–**T**). Perianth of Population No. 1–18, respectively; (**U**). Stamen of Population No. 1–18, respectively; Bar scale = 1 cm. Photo are taken in Thailand by Duangjai Tungmunnithum.

The distribution map of the collected 18 *N. nucifera* populations throughout Thailand is provided in Figure 1. According to the distribution of these 18 populations of this lotus species (Table 1 and Figure 1 and Figure 2), the most abundant floristic region in Thailand is the Central floristic region, where five populations of *N. nucifera* were found. The second most abundant floristic regions belong to Northern and North-Eastern floristic regions, where 4 populations of *N. nucifera* were found. The botanical description of *N. nucifera* (Nelumbonaceae family) used in this study is provide in the paragraph below.

Aquatic perennial, rhizomatous. Petiole 1–2.3 m, terete, glabrous or papillae hard and scattered. Leaf orbicular, blue-green, 28.5–90.5 cm in diam., glabrous, glaucous, water-repellent, margin entire. Flowers 9.5–24.5 cm in diam. Peduncles longer than petioles, glabrous or sparsely spinulate. Perianth caducous, oblong, oblong-elliptic or obovate, pink or white, 4.5–10.5 × 2.7–5.5 cm. Stamens longer than receptacle, filament slender; anther linear, 1–2.5 mm; connective appendage. Receptacle accrescent, turbinate, 4.5–9.5 cm in diam. Ovary Superior. Fruit oblong or ovoid, 1.3–2.5 × 6.5–15 cm, glabrous, pericarp thick, hardened.

Specimens examined: Stamens and perianths of *N. nucifera* populations #1–18.

### 2.2. Phytochemical Characterization

The total phenolic and flavonoid contents, as well as monomeric anthocyanin contents (TPC, TFC, and MAC, respectively) found in stamen (S) and perianth (P) extracts of *N. nucifera* populations from across Thailand’s floristic regions ranged from single to double, or even triple for the TFC of stamen extracts, demonstrating the high heterogeneity in phenolics/polyphenols accumulations observed for these medicinal species’ organs, and the importance to evaluate these contents prior to their use for further studies (e.g., determination of a biological activity) and/or industrial applications (Table 2).

The TPC ranged from 95.2 (P#10) to 233.9 (P#18) mg/100 g DW gallic acid equivalent for perianth extracts, and from 492.1 (S#5) to 669.4 (S#16) mg/100 g DW gallic acid equivalent for stamen extracts. The TFC ranged from 128.1 (P#12) to 315.7 (P#1) mg/100 g DW quercetin equivalent for perianth extracts, and from 570.6 (S#4) to 1,684.3 (S#14) mg/100 g DW quercetin equivalent for stamen extracts. The MAC ranged from 4.21 (P#9) to 8.89 (P#18) mg/100 g DW cyanidin-3-*O*-glucoside equivalent for perianth extracts, and from 1.37 (S#12) to 2.59 (S#1) mg/100 g DW cyanidin-3-*O*-glucoside equivalent for stamen extracts. From these results, the stamen appeared as a starting material high in TPC and TFC, although perianth was higher in MAC. 

**Figure 3:**
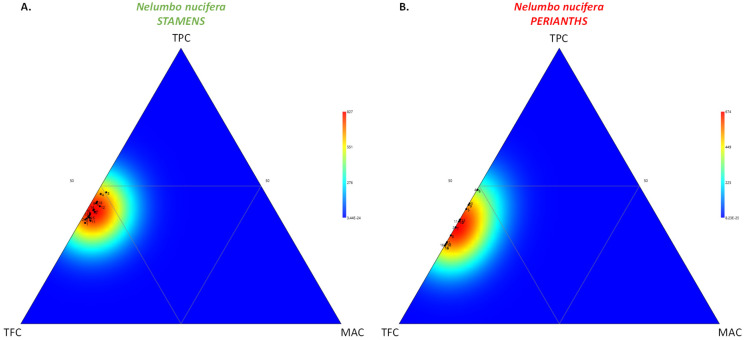
Ternary plot showing the visualization of the relative proportion of TPC, TFC, and TAC within the stamen (**A**) and perianth (**B**) extracts of 18 *N. nucifera* populations originating from various floristic regions from Thailand.

The significance of TFC as a key contributor to phytochemical variation was established in a ternary plot, as illustrated by the heatmap distribution, which was markedly shifted in the first bottom triangle (high TFC) for each *N. nucifera* organ (Figure 3).

Compared to other organs, few studies have dealt with the TPC, TFC, or MAC of stamen and perianth extracts from *N. nucifera*, but the observed variation ranges are consistent with the few published data [5,12,27,30,31,32,33]. Our results confirmed prior observations indicating that their accumulation varies substantially from one organ to another [10,27,28,30,32,34,35]. However, it is worth noting that, here, we described for the first time that there is a significant correlation (particularly so for TFCs) between the contents in perianth extract vs stamen extract for each class of phytochemicals. This indicates that, regardless of the organ, the accumulation capacity for a given population in a particular environment remains stable. This is highly intriguing since, in terms of minimum biomass, a rapid evaluation with perianth is easier to perform than with stamen. The relative impacts of these populations’ genetic backgrounds and the environment will be fascinating to assess over multiple years on the same site and with a specific population, or using the culture of descendants of the same population on other cultivation sites. Environmental factors, such as climatic and geographic (including growing conditions) factors, in addition to heredity, are well recognized to have a considerable impact on the accumulation of phenolic compounds [36,37].

**Figure 4:**
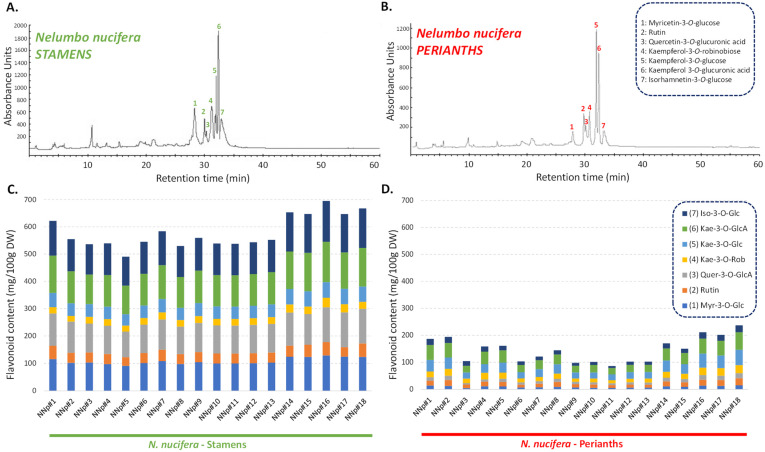
HPLC chromatograms (recorded at 320 nm) of the the stamen (**A**) and perianth (**B**) extracts of 18 *N. nucifera* populations originating from various floristic regions from Thailand. Quantification of the main flavonoids in the stamen (**C**) and perianth (**D**) extracts of 18 *N. nucifera* populations originating from various floristic regions from Thailand. Myr-3-*O*-Glc: myricetin-3-*O*-glucose; Quer-3-*O*-GlcA: quercetin-3-*O*-glucuronic acid; Kae-3-*O*-Rob: kaempferol-3-*O*-robinobiose; Kae-3-*O*-Glc: kaempferol-3-*O*-glucose; Kae 3-*O*-GlcA: kaempferol 3-*O*-glucuronic acid; Iso-3-*O*-Glc: isorhamnetin-3-*O*-glucose; the mean and standard deviation of the concentration of each flavonoid is provided in Table S1.

As our results identified TFC as the main phytochemical class of these stamen and perianth extracts, as well as key contributors to the observed variations, HPLC analyses were performed to offer a comprehensive understanding of the qualitative and quantitative changes (Figure 4, Table S1).

Individual flavonoid concentrations varied from 5.0 (myricetin-3-*O*-glucose, P#3) to 149.9 (isorhamnetin-3-*O*-glucose, S#16) mg/100 g DW in *N. nucifera* extracts. This analysis confirmed that stamen extracts are richer in flavonoids than perianth extracts. At the population level, the flavonoid content of stamen extracts is fairly balanced, with the predominant flavonoids being myricetin-3-*O*-glucose, quercetin-3-*O*-glucuronic acid, kaempferol 3-*O*-glucuronic acid, and isorhamnetin-3-*O*-glucose. Perianth extracts, on the other hand, accumulated mostly kaempferol derivatives, such as kaempferol-3-*O*-robinobiose and kaempferol-3-*O*-glucose (Figure 4, Table S1). The concentrations found here are comparable to those described by Temviriyanukul et al. [27] for aglycones generated by acidic hydrolysis. The occurrence of the three keampferol glycosides has been previously reported, but no quantification has been performed by the authors [12]. The present study is therefore the first to quantify different flavonoids accumulated in *N. nucifera* stamen and perianth extracts under their native glycosidic forms, which were investigated here at the population level.

**Figure 5:**
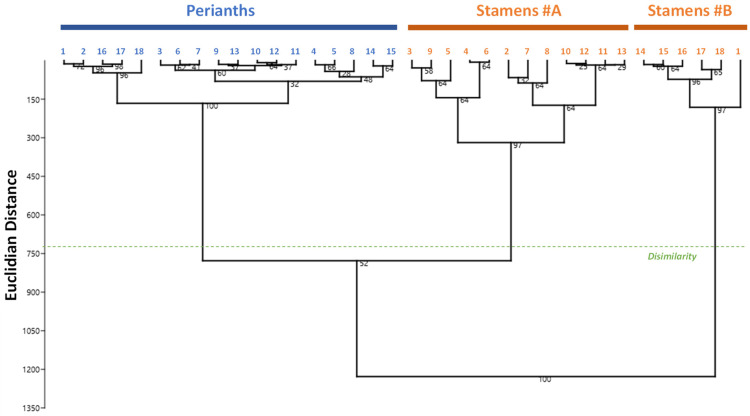
Hierarchical clustering analysis dendrogram according to the phytochemical composition of the stamen and perianth extracts of 18 *N. nucifera* populations originating from various floristic regions from Thailand. The percentages of replicate trees in which associated samples cluster together in the bootstrap test (percentage of 5000 replicates) are indicated next to the branches.

Hierarchical clustering analysis (HCA) was applied to identify potential groupings among the diverse samples from the various populations (Figure 5).

The hierarchical cluster analysis (HCA) revealed that the clustering occurred primarily at the organ level based on their phytochemical profiles. Perianth extracts were clustered together, whereas stamen extracts were divided into two subgroups based on their different flavonoid concentrations, with cluster stamen#B containing populations #1 and #14–18 being richer in flavonoids. However, no discernable pattern exists to demonstrate the significance of the genetic background factor. Considering the wide geographic distribution of the different *N. nucifera* populations across Thailand’s many floristic areas, environmental variables may explain at least some part of the heterogeneity in phytochemical profiles observed. 

Altogether, the present results provide a complete picture of the wide phytochemical—particularly flavonoid—variations observed at the organ level in different *N. nucifera* populations from the floristic regions of Thailand. Because we anticipate that these variations may have an impact on the health-promoting effect of *N. nucifera*, we next investigate how the antioxidant capacity of these extracts varies. Indeed, flavonoids have been shown to have a variety of health-promoting effects through their antioxidant activity [38].

### 2.3. Antioxidant Activity

The antioxidant capacity of the stamen and perianth extracts from the different *N. nucifera* populations to scavenge free radicals was evaluated using two major antioxidant mechanisms: the hydrogen atom transfer (HAT) mechanism evaluated with the ABTS assay, the single electron transfer (SET) mechanism evaluated with the FRAP assay, and the DPPH assay that allowed for the evaluation of both mechanisms [39,40]. The results are summarized in Table 3.

**Figure 6:**
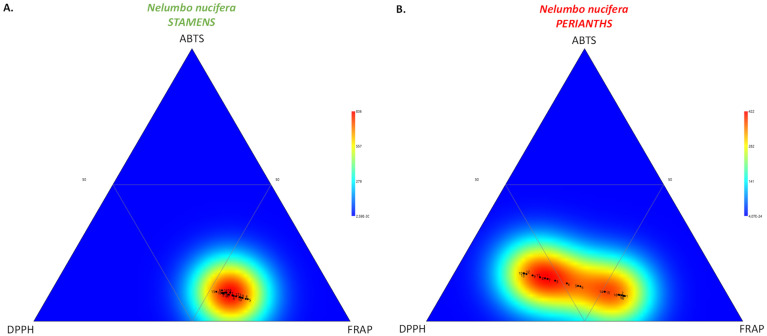
Ternary plot showing the visualization of the relative contribution of the different in vitro assays (ABTS, DPPH and FRAP) to the antioxidant capacitiy of the stamen (**A**) and perianth (**B**) extracts of 18 *N. nucifera* populations originating from various floristic regions from Thailand.

Here (Table 3), the ABTS radical scavenging activity ranged from 24.0 (P#18) to 40.4 (P#10) µmol TE/g DW for the perianth extracts, and from 27.4 (S#6) to 60.5 (S#15) µmol TE/g DW for the stamen extracts. The DPPH free radical scavenging activity ranged from 85.3 (P#18) to 139.3 (P#10) µmol TE/g DW for the perianth extracts, and from 96.4 (S#6) to 204.5 (S#18) µmol TE/g DW for the stamen extracts. The FRAP reducing power ranged from 49.3 (P#12) to 162.7 (P#2) µmol TE/g DW for the perianth extracts, and from 210.0 (S#6) to 319.6 (S#16) µmol TE/g DW for the stamen extracts. The present results revealed the importance of the HAT antioxidant mechanism over the ET mechanism, as shown in Figure 6, with the FRAP assay for stamen extract (Figure 6A) and both FRAP and DPPH assays for perianth extracts contributing the most to their antioxidant capacity (Figure 6B).

Our results are consistent with the work of Jung et al. [12], who showed that flavonoids from *N. nucifera* stamen extract, particularly the kaempferol glycosides, exhibit strong radical scavenging capacity. The observed prominence of the HAT mechanism is consistent with the observation that flavonoids prefer HAT-based reactions to ET-based reactions when quenching free radicals [40,41,42]. In contrast, Temviriyanukul et al. [27] reported that the ET mechanism contributed the most to the antioxidant capacity of their *N. nucifera* extract. Other compounds, particularly phenolics [40,41], which contribute mainly through an ET-based mechanism, may also contribute to this antioxidant action. In support of our results, Lin et al. [8] showed that flavonoids mainly contributed to antioxidant activities in *N. nucifera* compared to other phenolics.

### 2.4. Correlation Analysis

**Figure 7:**
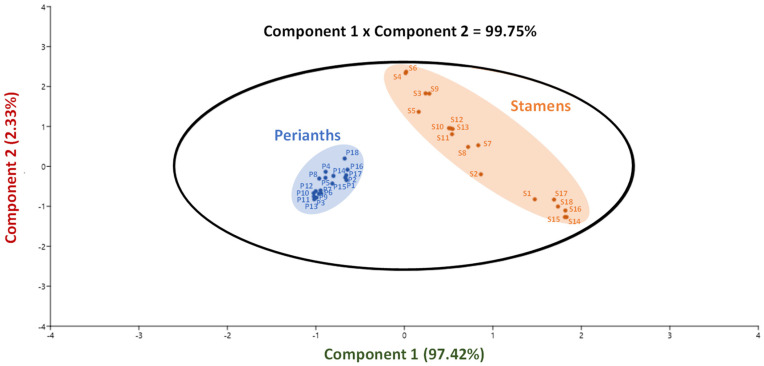
Principal component analysis (PCA) linking the phytochemical profile and antioxidant capacity of the stamen and perianth extracts of 18 *N. nucifera* populations originating from various floristic regions from Thailand. Variance of component 1 = 97.42% and component = 2.33%. S: stamen extract; P: perianth extract; each number in blue represents the different populations. The corresponding loading score plots for components 1 and 2 are presented in Figure S1.

Different variables were used in a principal component analysis (PCA) to extract relevant connections between the metabolic composition and antioxidant activities within the stamen and perianth extracts from different *N. nucifera* natural populations (Figure 7). 

The generated biplot representation explained 99.75% of the initial variability (Figure 5). The TFC and the FRAP antioxidant assay are the key contributing elements to the discrimination along the component 1 axis, which accounts for 97.42% of the initial variability (Figure S1). The second component axis, on the other hand, accounted for only 2.33%. As a consequence of this PCA, two different clusters were shown to be significantly different from one another based on their TFC and FRAP antioxidant activity. Remarkably, these clusters distinguished stamen extracts, which are high in flavonoids and have high FRAP antioxidant activity, from perianth extracts, which contribute less to both parameters. This study demonstrated the value of stamen as a starting material for applications based on antioxidant flavonoids derived from *N. nucifera*.

Pearson correlation coefficients (PCC) were calculated to assess the relationship between each phytochemical and antioxidant activity (Table 4).

This correlation analysis clearly established the strength of the association between TPC, TFC (including each individual flavonoid), and MAC and the several antioxidant assays. Significant correlations between various classes of phytochemicals and the different antioxidant tests were noted. This emphasized the fact that the antioxidant capability of an extract is generally the result of complex phytochemical combinations rather than the action of a single molecule [43]. Considering the importance of the HAT antioxidant mechanism to the antioxidant capacity of the *N. nucifera* extracts, the strongest and most significant correlations linking TPC, TFC, as well as myricetin-3-*O*-glucose, rutin, quercetin-3-*O*-glucuronic acid, kaempferol-3-*O*-glucuronic acid, and isorhamnetin-3-*O*-glucose content to the FRAP assay are certainly more notable. The specific contributions of the kaempferol glycosides isolated from *N. nucifera* stamen to the strong radical scavenging capacity of this extract have already been resolved [12]. The present correlation analysis emphasized that the roles of the other flavonoids should also be investigated. 

## 3. Materials and Methods

### 3.1. Chemicals and Reagents

The solvents and reagents for extraction as well as for HPLC analysis were of analytical grade or the highest available purity (Thermo Fischer Scientific, Illkirch, France). The deionized water was purified using the Milli-Q water-purification system (Merck Millipore Fontenay sous Bois, Paris, France). All of the prepared solutions for HPLC were filtered through 0.45-µm nylon syringe membranes before use. The standards were purchased from Extrasynthese (Genay, France). 

### 3.2. Plant Materials

The living plant materials of 18 *N. nucifera* populations were collected from the floristic regions in Thailand, including Northern (N), North-Eastern (NE), Central (C), South-Eastern (SE), Eastern (E), South-Western (SW), and Peninsula (PEN). After reviewing the literature as well as information about the *N. nucifera* herbarium specimens, the targeted populations in various localities covering every floristic region in the country were searched, in order to find living specimens in the fields. The collected plant samples were identified at the species level using the taxonomic key and description in the existing Floras [1,2], and compared with the herbarium specimens kept at Forest Herbarium (BKF), Bangkok, Thailand, by Prof. Kasin Suvatabandhu from Herbarium, Chulalongkorn University, (BCU). Herbarium abbreviations are used according to Thiers [44]. The stamen and perianth from 18 populations of *N. nucifera* were air-dried and prepared following World Health Organization recommendations [45]. 

### 3.3. Extraction 

The dried stamen or perianth samples (100 mg/sample) were placed in 5 mL quartz tubes equipped with a vapor condenser, then extracted by ultrasound-assisted extraction in 1 mL 90% (*v*/*v*) aqEtOH in the USC1200TH ultrasonic bath (Prolabo, Fontenay-sous-Bois, France) following the optimized extraction conditions: 30 kHz frequency at 45 °C for 45 min [33]. Then, the extract was centrifuged for 15 min at 5000× *g* (Heraeus Biofuge Stratos, Thermo Scientific, Illkirch, France). The obtained supernatant was filtered using 0.45-μm nylon syringe membranes (Merck Millipore, Saint-Quentin Fallavier, France). Flavonoid enrichment was then performed through the additional DAX-8 (Merck Millipore, Saint-Quentin Fallavier, France) macroporous resin purification step as described in the previously study [33].

### 3.4. Determination of Total Phenolic Content (TPC) 

The TPC was measured following the Folin–Ciocalteu protocol and microplate spectrophotometry, as previously described by Tungmunnithum et al. [46]. Absorbance was then measured at 650 nm using a spectrophotometer (BioTek ELX800 Absorbance Microplate Reader, BioTek Instruments, Colmar, France). After that, the standard curve (0–40 µg/mL; R^2^ = 0.998) of gallic acid (Merck, Saint-Quentin Fallavier, France) was employed to express the total phenolic content in mg of gallic acid equivalents/g DW (mg GAE/100 g dry weight (DW)).

### 3.5. Determination of Total Flavonoid Content (TFC) 

The TFC was examined following the colorimetric aluminum trichloride (AlCl_3_) method [33]. The 200 µL mixture was prepared in a microplate using 20 µL of extract sample, 10 µL of potassium acetate 1 M, 10 µL of AlCl_3_ (10% (*w*/*v*)), as well as 160 µL of deionized water. Subsequently, the microplate reader (Multiskan GO, Thermo Fischer Scientific, Illkirch, France) was employed to measure the absorbance at 415 nm after 30 min of incubation in the dark at 25 °C. The TFC was expressed in mg/100 g dry weight (DW) of quercetin equivalent using the five-point calibration line (linearity range from 0 to 40 g/mL quercetin concentration with the R^2^ of 0.998).

### 3.6. Determination of Total Anthocyanin Content (TAC) 

The TAC was determined using the colorimetric method following the previous study [47]. Absorbance was then measured at 510 and 700 nm using a spectrophotometer (BioTek ELX800 Absorbance Microplate Reader, BioTek Instruments, Colmar, France). The standard curve (0–100 µg/mL, R^2^ = 0.999) of cyanidin-3-*O*-glucoside (Merck, Saint-Quentin Fallavier, France) was employed to express the total anthocyanin content in mg of cyanidin-3-*O*-glucoside equivalents/ g DW (mg CAE/100 g DW).

### 3.7. High-Performance Liquid Chromatography (HPLC) Analysis 

The high-performance liquid chromatography system composed of the autosampler, Varian (Les Ulis, France) Prostar 230 pump, as well as the Varian Prostar 335 photodiode array detector was used to analyze and controlled with the Galaxie software (Varian v1.9.3.2). The separation was then carried out on the Purospher RP-18 column (250 × 4.0 mm internal diameter; 5 µm) (Merck Chemicals, Molsheim, France) at 40 °C. The mobile phase consisted of the mixture of methanol (solvent A) and HPLC grade water (solvent B), which were acidified with 0.05% formic acid. Then, the linear gradient, from 0 to 60 min was applied to this mobile phase variation, ranging from the 5:95 (*v*/*v*) to 100:0 (*v*/*v*) mixture of solvents A and B, respectively, using a flow rate of 1.30 mL/min. The injection volume was 3 µL, and the maximum back pressure was 110 bar. Detection was performed at 320 nm. The flavonoid compounds were identified by comparison with authentic standards (Sigma Aldrich).

### 3.8. In Vitro Cell Free Antioxidant Assays 

The in vitro cell free antioxidant assays, including DPPH (2,2-diphenyl-1-picrylhydrazyl), FRAP (Ferric Reducing Antioxidant Power) as well as ABTS (2,2-azinobis(3-ethylbenzthiazoline-6-sulphonic acid) assays, were examined to evaluate the antioxidant activity of the extract samples following the protocols adapted to the microplate reader (Multiskan GO, Thermo Fischer Scientific, Illkirch, France), as previously described [33,36].

### 3.9. Statistical Analysis 

Statistical analyses were performed using the XLSTAT 2019 suite (Addinsoft, Paris, France) and the PAST4.0 [48]. The data composed of at least the three independent replicates were presented in the form of mean and standard deviation. The Student’s t-test was performed for statistical comparative analysis. Significant differences at *p* < 0.05, 0.01 as well as 0.001 were presented using *, **, and ***, respectively. Different letters were employed to indicate the significant thresholds at *p* < 0.05.

## 4. Conclusions

To recapitulate, the studied 18 populations of *N. nucifera* obtained from their natural habitats throughout the floristic regions of Thailand displayed a high heterogeneity in terms of their phenolics/polyphenols accumulations observed in perianth and stamen. Furthermore, this analysis showed that flavonoids are the main phytochemical class of these extracts, and that the stamen is richer in flavonoids than perianth. Besides, this is also the first description of the significant correlation between the phytochemical contents in perianth and stamen extracts. In addition, the in vitro, cell-free antioxidant approaches point out that the antioxidant capacity of these *N. nucifera* populations is mainly mediated by a hydrogen atom transfer mechanism. The current finding also emphasized that the antioxidant potential of the extracts is the result of the complex phytochemical combinations as opposed to a single molecule. This present research offers frontier knowledge on the phytochemical diversity and antioxidant potential of the perianth and stamen from *N. nucifera* natural populations covering all floristic regions of Thailand, to open the door for phytopharmaceutical sectors seeking potential raw plant material/plant organs to design and develop their different products.

## Figures and Tables

**Table 1:** The collected 18 populations of *N. nucifera* throughout Thailand.

Floristic Regions	Population No.	Collected Locations/Provinces	Collected Months	Collected Season
Northern (N)	1	Sukhothai	April	Summer
	2	Lampang	May	Summer
	3	Phitsanulok	August	Rainy
	4	Nakhon Sawan	September	Rainy
North-Eastern (NE)	5	Udon Thani	November	Winter
	6	Khon Kaen	April	Summer
	7	Nong Khai	September	Rainy
	8	Roi Et	November	Winter
South-Western (SW)	9	Kanchanaburi	April	Summer
	10	Ratchaburi	September	Rainy
Central (E)	11	Suphan Buri	November	Winter
	12	Angthong	Decemember	Winter
Eastern (E)	13	Phra NakhonSi Ayuthaya	May	Summer
	14	Pathum Thani	October	Rainy
	15	Nakhon Pathom	April	Summer
	16	Nakhon Ratchasima	September	Rainy
South-Eastern (SE)	17	Chachoengsao	April	Summer
Peninsular (PEN)	18	Phatthalung	September	Rainy

**Table 2:** Phytochemical profiles of stamen and perianth extracts from 18 *N. nucifera* populations originating from various floristic regions from Thailand.

Sample	TPC (mg/100 g DW)	TFC (mg/100 g DW)	MAC (mg/100 g DW)
S#1	621.6 ± 1.5 ^b^	1474.3 ± 35.2 ^c^	2.47 ± 0.14 ^ab^
S#2	548.7 ± 4.0 ^de^	1019.5 ± 69.3 ^d^	2.59 ± 0.11 ^a^
S#3	553.8 ± 4.5 ^de^	712.3 ± 35.9 ^f^	1.69 ± 0.10 ^c^
S#4	542.5 ± 16.4 ^de^	570.6 ± 1.7 ^g^	1.69 ± 0.02 ^c^
S#5	492.1 ± 19.1 ^f^	688.5 ± 74.1 ^f^	1.63 ± 0.02 ^c^
S#6	544.7 ± 4.3 ^e^	575.4 ± 3.1 ^g^	1.56 ± 0.08 ^cd^
S#7	580.7 ± 5.5 ^c^	1075.7 ± 0.7 ^d^	1.59 ± 0.04 ^c^
S#8	529.4 ± 7.8 ^e^	1119.5 ± 78.3 ^d^	1.45 ± 0.04 ^de^
S#9	559.4 ± 1.3 ^d^	736.4 ± 60.7 ^f^	1.51 ± 0.01 ^d^
S#10	538.6 ± 24.5 ^def^	887.4 ± 24.1 ^e^	1.42 ± 0.01 ^e^
S#11	537.7 ± 6.4 ^d^	909.9 ± 78.3 ^e^	1.45 ± 0.04 ^d^
S#12	543.2 ± 33.6 ^cdef^	897.8 ± 4.1 ^e^	1.37 ± 0.04 ^e^
S#13	551.9 ± 1.1 ^e^	904.7 ± 81.4 ^ef^	1.56 ± 0.01 ^c^
S#14	667.5 ± 21.5 ^a^	1684.3 ± 34.8 ^a^	2.14 ± 0.06 ^b^
S#15	663.9 ± 14.2 ^a^	1673.3 ± 3.8 ^a^	2.11 ± 0.18 ^b^
S#16	670.7 ± 24.9 ^a^	1671.9 ± 7.2 ^a^	2.40 ± 0.08 ^a^
S#17	669.4 ± 30.6 ^a^	1590.9 ± 4.1 ^b^	2.57 ± 0.08 ^a^
S#18	664.0 ± 18.8 ^a^	1620.6 ± 40.7 ^ab^	2.25 ± 0.06 ^b^
P#1	187.6 ± 7.3 ^a^	315.7 ± 4.1 ^a^	9.06 ± 0.34 ^a^
P#2	189.2 ± 1.3 ^a^	305.2 ± 2.6 ^b^	5.90 ± 0.71 ^d^
P#3	108.1 ± 24.0 ^de^	162.6 ± 15.2 ^e^	4.88 ± 0.46 ^de^
P#4	166.7 ± 19.8 ^abc^	182.3 ± 1.7 ^d^	4.97 ± 0.61 ^de^
P#5	152.8 ± 4.9 ^c^	187.4 ± 6.2 ^d^	5.67 ± 0.10 ^d^
P#6	112.0 ± 38.5 ^cd^	176.8 ± 11.4 ^de^	4.32 ± 0.22 ^e^
P#7	120.9 ± 4.3 ^d^	168.5 ± 1.0 ^e^	8.33 ± 0.93 ^ab^
P#8	143.5 ± 2.3 ^c^	148.8 ± 6.2 ^e^	8.45 ± 2.57 ^abcd^
P#9	111.1 ± 1.9 ^d^	138.8 ± 9.3 ^ef^	4.21 ± 0.97 ^de^
P#10	95.2 ± 0.9 ^e^	131.2 ± 7.9 ^f^	4.66 ± 0.42 ^de^
P#11	86.7 ± 33.8 ^de^	138.1 ± 2.4 ^f^	7.08 ± 0.12 ^c^
P#12	101.2 ± 24.9 ^de^	128.1 ± 5.5 ^f^	7.96 ± 2.32 ^abcd^
P#13	100.3 ± 6.2 ^de^	148.5 ± 2.4 ^e^	4.42 ± 0.40 ^e^
P#14	171.2 ± 6.4 ^b^	233.3 ± 0.3 ^c^	8.71 ± 0.28 ^ab^
P#15	152.1 ± 3.8 ^c^	234.3 ± 6.9 ^c^	8.73 ± 0.16 ^a^
P#16	214.4 ± 42.8 ^ab^	311.9 ± 1.0 ^ab^	8.41 ± 0.14 ^b^
P#17	201.7 ± 14.1 ^a^	310.2 ± 2.8 ^ab^	8.61 ± 0.14 ^ab^
P#18	233.9 ± 57.3 ^ab^	285.4 ± 17.2 ^ab^	8.89 ± 1.01 ^ab^

S: stamen; P: perianth; #i indicate the population number i; TPC: total phenolic content; TFC: total flavonoid content; MAC: monomeric anthocyanin content; DW: dry weigth. Different supercript letters indicate significant differences at *p* < 0.05.

**Table 3:** In vitro cell-free antioxidant activity of the stamen and perianth extracts of 18 *N. nucifera* populations originating from various floristic regions from Thailand.

Sample	ABTS (µmol TEAC)	DPPH (µmol TEAC)	FRAP (µmol TEAC)
S#1	54.2 ± 2.4 ^bc^	185.0 ± 7.9 ^b^	291.5 ± 2.6 ^b^
S#2	43.2 ± 1.2 ^d^	148.6 ± 4.0 ^c^	265.8 ± 2.5 ^d^
S#3	33.1 ± 0.4 ^gh^	115.1 ± 1.3 ^ef^	227.7 ± 5.5 ^g^
S#4	33.6 ± 1.2 ^gh^	116.9 ± 4.0 ^ef^	213.0 ± 0.7 ^h^
S#5	29.9 ± 1.6 ^ij^	104.8 ± 5.3 ^gh^	235.4 ± 5.4 ^g^
S#6	27.4 ± 0.4 ^j^	96.4 ± 1.3 ^h^	210.0 ± 2.3 ^h^
S#7	53.7 ± 10.4 ^abcd^	183.1 ± 34.3 ^abc^	281.0 ± 5.8 ^bc^
S#8	59.0 ± 2.8 ^ab^	182.1 ± 50.1 ^abcd^	281.6 ± 4.3 ^c^
S#9	31.6 ± 1.8 ^hi^	110.4 ± 5.8 ^fg^	229.4 ± 5.6 ^g^
S#10	35.6 ± 1.6 ^fg^	123.5 ± 5.7 ^e^	249.8 ± 3.8 ^ef^
S#11	42.4 ± 1.5 ^de^	145.8 ± 5.3 ^cd^	246.6 ± 0.3 ^f^
S#12	39.0 ± 2.4 ^ef^	134.6 ± 7.9 ^de^	253.9 ± 3.2 ^e^
S#13	52.0 ± 2.9 ^c^	177.5 ± 7.9 ^b^	251.4 ± 6.7 ^ef^
S#14	60.2 ± 0.4 ^a^	195.9 ± 11.5 ^ab^	317.3 ± 14.0 ^a^
S#15	60.5 ± 0.8 ^a^	202.0 ± 8.9 ^ab^	319.6 ± 13.1 ^a^
S#16	58.8 ± 1.6 ^ab^	183.7 ± 4.8 ^b^	319.5 ± 18.9 ^a^
S#17	59.3 ± 0.8 ^a^	192.8 ± 18.3 ^ab^	317.1 ± 21.1 ^ab^
S#18	60.2 ± 0.4 ^a^	204.5 ± 1.3 ^a^	316.3 ± 18.6 ^ab^
P#1	25.1 ± 0.6 ^g^	89.0 ± 1.7 ^f^	158.4 ± 5.7 ^a^
P#2	27.1 ± 0.8 ^e^	95.5 ± 2.6 ^g^	162.7 ± 7.5 ^a^
P#3	31.4 ± 2.0 ^cd^	109.5 ± 6.6 ^cd^	70.2 ± 7.3 ^ef^
P#4	27.7 ± 1.8 ^def^	97.4 ± 5.3 ^e^	91.6 ± 16.9 ^ef^
P#5	27.1 ± 1.6 ^defg^	95.5 ± 5.8 ^ef^	86.8 ± 1.8 ^f^
P#6	28.5 ± 2.0 ^de^	100.2 ± 6.6 ^de^	77.1 ± 4.8 ^e^
P#7	32.8 ± 1.6 ^c^	114.1 ± 5.3 ^c^	64.6 ± 6.1 ^gh^
P#8	33.3 ± 0.8 ^c^	116.0 ± 2.6 ^c^	62.9 ± 2.1 ^g^
P#9	36.7 ± 3.2 ^abc^	127.2 ± 10.5 ^abc^	55.1 ± 1.5 ^hi^
P#10	40.4 ± 0.4 ^a^	139.3 ± 1.3 ^a^	50.5 ± 2.6 ^i^
P#11	33.1 ± 0.7 ^c^	115.1 ± 1.5 ^c^	57.2 ± 2.3 ^hi^
P#12	37.0 ± 1.2 ^b^	128.1 ± 4.0 ^b^	49.3 ± 2.1 ^i^
P#13	39.8 ± 2.8 ^ab^	137.4 ± 9.2 ^ab^	50.6 ± 3.1 ^i^
P#14	25.4 ± 0.8 ^fe^	89.9 ± 2.6 ^f^	119.4 ± 4.6 ^d^
P#15	25.1 ± 0.4 ^g^	89.0 ± 1.3 ^f^	118.8 ± 3.5 ^d^
P#16	25.4 ± 0.8 ^hg^	89.9 ± 2.6 ^f^	157.2 ± 4.4 ^a^
P#17	24.6 ± 0.4 ^g^	87.1 ± 1.1 ^f^	144.0 ± 3.2 ^b^
P#18	24.0 ± 0.3 ^g^	85.3 ± 1.7 ^f^	138.3 ± 0.9 ^c^

S: stamen; P: perianth; #i indicate the population number i; ABTS: 2,2-azinobis (3-ethylbenzthiazoline-6-sulphonic acid; DPPH: 2,2-diphenyl-1-picrylhydrazyl; FRAP: ferric reducing antioxidant power. Different supercript letters indicate significant differences at *p* < 0.05.

**Table 4:** Pearson coefficient correlation) between phytochemical profiles and antioxidant activities of the stamen and perianth extracts of 18 *N. nucifera* populations.

Compound	ABTS	DPPH	FRAP
TPC	0.699 ***	0.698 ***	0.969 ***
TFC	0.860 ***	0.853 ***	0.941 ***
MAC	−0.609 ***	−0.616 ***	−0.710 ***
Myr-3-*O*-Glc	0.719 ***	0.719 ***	0.950 ***
Rutin	0.666 ***	0.668 ***	0.957 ***
Quer-3-*O*-GlcA	0.704 ***	0.705 ***	0.946 ***
Kae-3-*O*-Rob	0.338 *	0.310 ns	0.753 ***
Kae-3-*O*-Glc	0.453 **	0.442 **	0.873 ***
Kae-3-*O*-GlcA	0.681 ***	0.678 ***	0.977 ***
Iso-3-*O*-Glc	0.716 ***	0.716 ***	0.955 ***

*** significant *p* < 0.001; ** significant *p* < 0.01; * significant *p* < 0.05; ns: not significant.

## Data Availability

All the data supporting the findings of this study are included in this article.

## Supplementary Materials

The following supporting information can be downloaded, Figure S1: Loading scores of the component 1 and component 2 of the PCA (presented in Figure 5) linking the phytochemical profile and antioxidant capacity of the stamen and perianth extracts of 18 *N. nucifera* populations originating from various floristic regions from Thailand; Table S1: HPLC quantification of the main flavonoids in the stamen (A) and perianth (B) extracts of 18 *N. nucifera* populations originating from various floristic regions from Thailand.

## Sample Availability

Samples of the compounds are available from the authors.
