# Supplementary material for: Phytochemical Diversity and Antioxidant Potential of Natural Populations of Nelumbo nucifera Gaertn. throughout the Floristic Regions in Thailand
Source: Molecules. 2022 Jan 20;27(3):681. doi: 10.3390/molecules27030681 (PMC8840423; doi:10.3390/molecules27030681)
Supplement: Supplementary file 1 [file molecules-27-00681-s001.zip › molecules-1561339-supplementary.pdf]

Supplementary Materials

Phytochemical Diversity and Antioxidant Potential of Natural Populations of *Nelumbo nucifera* Gaertn. throughout the Floristic Regions in Thailand

Duangjai Tungmunnithum [1,2,3,*], Samantha Drouet [2] and Christophe Hano [2,3,*]

1 Department of Pharmaceutical Botany, Faculty of Pharmacy, Mahidol University, Bangkok 10400, Thailand
2 Laboratoire de Biologie des Ligneux et des Grandes Cultures, INRAE USC1328, Campus Eure et Loir,
Orleans University, 28000 Chartres, France; samantha.drouet@univ-orleans.fr
3 Le Studium Institue for Advanced Studies, 1 Rue Dupanloup, 45000 Orléans, France
* Correspondence: duangjai.tun@mahidol.ac.th (D.T.); hano@univ-orleans.fr (C.H.)

Table S1. HPLC quantification of the main flavonoids in the stamen (A) and perianth (B) extracts of 18 *N. nucifera* populations originating from various floristic regions from Thailand.

Sample	*(1) Myr-3-O-Glc*		*(2) Rutin*		*(3) Quer-3-O-GlcA*		*(4) Kae-3-O-Rob*		*(5) Kae-3-O-Glc*		*(6) Kae-3-O-GlcA*		*(7) Iso-3-O-Glc*	
	mean	SD	mean	SD	mean	SD	mean	SD	mean	SD	mean	SD	mean	SD
S#1	115.6	6.0	48.6	6.9	118.1	17.6	22.9	1.2	52.6	2.9	136.5	7.6	127.4	18.5
S#2	102.0	0.1	36.7	0.1	114.3	0.3	20.6	0.0	46.4	0.2	117.0	0.5	117.7	0.4
S#3	103.0	1.5	37.0	1.5	105.2	4.4	24.8	0.4	46.8	3.2	108.1	6.1	110.8	4.5
S#4	97.9	0.4	36.3	0.4	103.1	1.3	24.3	0.1	45.9	1.0	115.6	2.0	116.4	1.5
S#5	91.5	2.6	30.9	2.4	93.5	7.7	22.1	0.6	41.6	5.6	104.9	11.7	105.6	8.4
S#6	101.3	12.1	36.4	12.0	103.5	15.8	24.4	2.9	46.1	26.3	116.1	14.6	116.9	9.5
S#7	108.0	0.2	41.8	0.2	110.4	0.5	26.0	0.0	49.1	0.4	123.8	0.8	124.6	0.6
S#8	98.5	0.8	35.4	0.8	100.6	2.5	23.7	0.2	44.8	1.8	112.9	3.7	113.6	2.7
S#9	104.0	2.9	37.4	2.9	106.3	8.7	25.1	0.7	47.3	6.4	119.3	13.3	120.0	9.6
S#10	100.2	3.4	36.0	3.4	102.3	10.1	24.1	0.8	45.5	7.4	114.8	15.4	115.6	11.2
S#11	100.0	10.5	36.0	10.4	102.2	10.9	24.1	2.5	45.5	2.3	114.6	17.1	115.4	4.1
S#12	101.0	6.3	36.3	6.2	103.2	18.5	24.4	1.5	45.9	3.6	115.8	8.3	116.5	20.4
S#13	102.6	2.6	36.9	2.6	104.9	7.8	24.7	0.6	46.7	5.7	117.7	11.9	118.4	8.6
S#14	124.1	0.2	40.6	0.2	120.8	0.6	29.9	0.0	56.4	0.5	137.3	0.9	143.2	0.7
S#15	123.5	1.0	44.4	1.0	112.2	2.7	27.8	0.2	56.1	2.3	140.5	4.7	142.5	3.4
S#16	128.7	3.6	48.8	3.8	127.4	10.4	35.1	1.0	56.7	7.7	148.0	16.5	149.9	12.0
S#17	124.5	3.5	34.8	2.7	127.2	10.4	30.0	0.8	56.6	7.6	132.7	14.8	140.6	11.2
S#18	123.5	14.8	49.4	16.3	126.2	13.6	25.8	3.0	56.1	2.0	141.6	16.5	144.5	8.8
P#1	14.1	1.9	18.2	4.9	13.1	3.9	21.0	2.1	42.0	1.9	55.4	0.7	22.7	6.4
P#2	13.3	0.7	21.3	4.2	16.2	3.3	25.2	1.8	41.4	4.0	53.9	5.0	22.9	4.6
P#3	5.0	0.5	12.6	3.4	5.7	1.6	16.9	1.6	23.0	0.6	24.1	3.0	16.9	4.6
P#4	11.8	1.0	16.9	4.0	8.4	2.1	24.4	2.0	32.0	4.1	45.8	4.9	19.4	4.3
P#5	10.9	1.0	16.4	3.8	12.3	3.0	22.6	1.9	36.7	4.6	45.1	4.9	16.9	4.0
P#6	7.3	0.3	10.0	1.8	9.0	1.7	14.4	0.9	22.9	7.5	27.1	2.5	12.4	2.3
P#7	7.8	0.6	13.0	2.8	9.7	2.2	15.5	1.2	29.0	1.3	32.5	3.3	13.3	3.3
P#8	9.3	0.6	18.4	3.4	11.5	2.2	19.4	1.3	34.5	1.0	35.6	3.1	15.8	3.0
P#9	7.2	0.5	11.9	2.1	8.9	1.6	12.2	0.8	22.7	2.9	24.9	2.1	10.3	1.8
P#10	6.2	0.5	12.2	2.5	7.6	1.6	14.2	1.0	22.9	4.0	27.6	2.7	10.5	2.2
P#11	5.6	0.5	10.3	2.4	7.0	1.7	11.1	0.9	21.8	8.6	23.3	2.5	7.6	1.8
P#12	6.6	0.4	10.9	1.9	9.1	1.6	13.0	0.8	24.3	7.2	27.2	2.2	11.2	2.0
P#13	6.5	0.5	10.8	2.1	8.0	1.7	14.9	1.0	24.1	8.2	27.0	2.5	11.1	2.2
P#14	11.1	0.4	18.4	3.2	13.7	1.7	21.9	3.7	41.1	4.8	45.0	2.2	18.9	2.1
P#15	9.9	0.1	16.4	1.4	12.2	0.3	19.5	1.2	35.5	2.8	40.9	0.7	15.8	0.2
P#16	13.9	1.5	22.0	2.8	17.2	2.5	27.5	3.0	51.5	6.3	55.6	2.0	23.7	3.0
P#17	13.1	0.5	21.7	1.0	16.2	0.8	27.8	1.1	46.4	2.1	54.2	0.7	22.3	1.0
P#18	15.1	1.1	26.2	5.3	18.8	4.0	30.0	2.2	56.2	9.8	64.9	2.9	25.8	5.4

Myr-3-O-Glc: myricetin-3-O-glucose; Quer-3-O-GlcA: quercetin-3-O-glucuronic acid; Kae-3-O-Rob: kaempferol-3-O-robinobiose; Kae-3-O-Glc: kaempferol-3-O-glucose; Kae 3-O-GlcA: kaempferol 3-O-glucuronic acid; Iso-3-O-Glc: isorhamnetin-3-O-glucose. Means and standard deviations of 3 independent analysis.

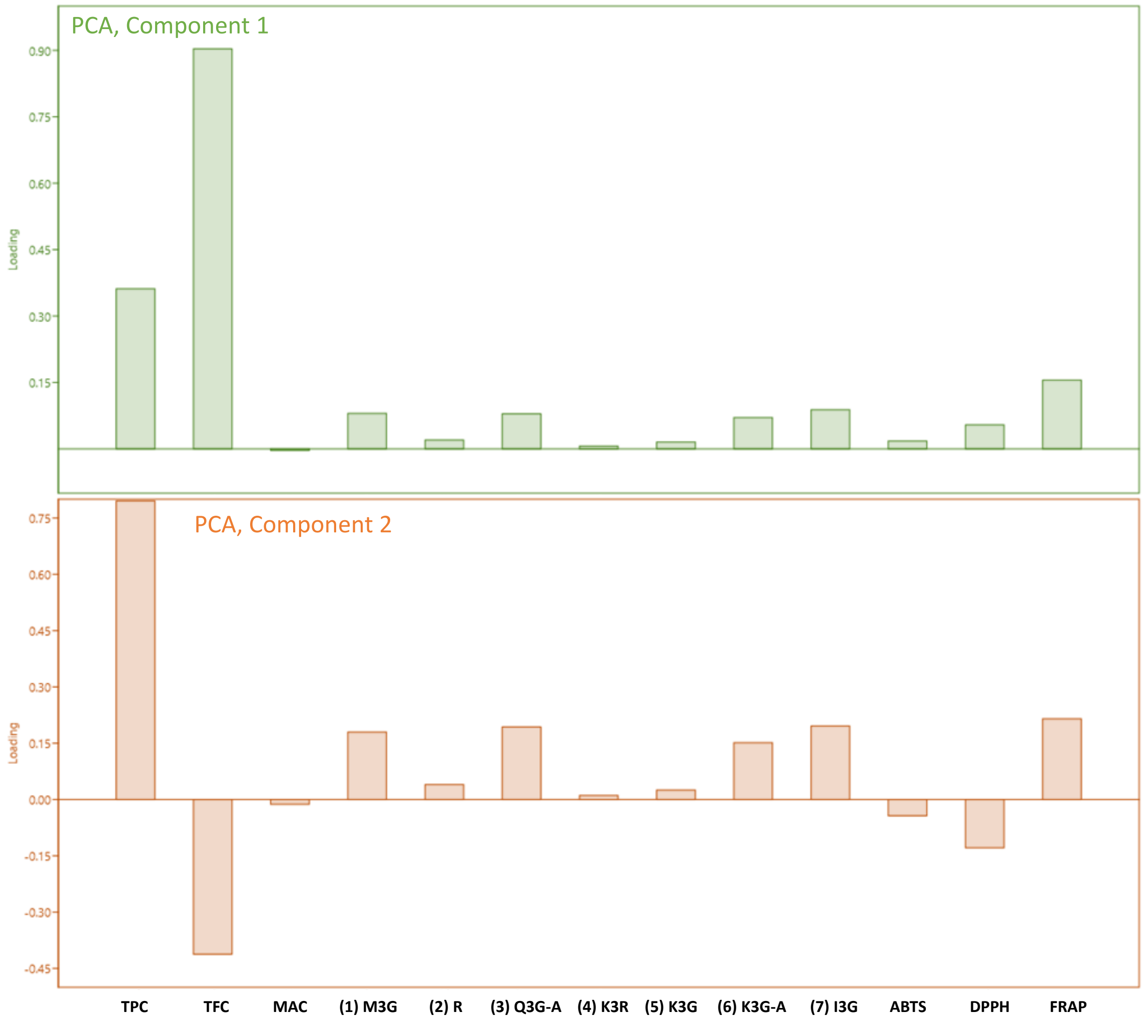

Figure S1. Loading scores of the component 1 and component 2 of the PCA (presented in Figure 5) linking the phytochemical profile and antioxidant capacity of the stamen and perianth extracts of 18 *N. nucifera* populations originating from various floristic regions from Thailand.
